# 多谱系累及Ph阳性急性淋巴细胞白血病的诊疗研究进展

**DOI:** 10.3760/cma.j.cn121090-20250228-00099

**Published:** 2025-07

**Authors:** 德麟 卢, 琦敏 张, 乐 李, 闰夏 顾

**Affiliations:** 1 中国医学科学院血液病医院（中国医学科学院血液学研究所），血液与健康全国重点实验室，国家血液系统疾病临床医学研究中心，细胞生态海河实验室，天津 300020 State Key Laboratory of Experimental Hematology, National Clinical Research Center for Blood Diseases, Haihe Laboratory of Cell Ecosystem, Institute of Hematology & Blood Diseases Hospital, Chinese Academy of Medical Sciences & Peking Union Medical College, Tianjin 300020, China; 2 天津医学健康研究院，天津 301600 Tianjin Institutes of Health Science, Tianjin 301600, China

## Abstract

基于分子遗传学改变和微小残留病（MRD）监测的动态分层治疗体系，为Ph染色体阳性急性淋巴细胞白血病（Ph^+^ ALL）的临床管理奠定了坚实基础。然而，随着免疫靶向治疗的应用推广和检测灵敏度的不断提升，临床上逐渐观察到部分Ph^+^ ALL患者存在多种方法MRD检测结果不匹配的现象，尤其是基于BCR::ABL1的MRD检测结果持续高于其他检测指标。深入研究表明，BCR::ABL1的持续阳性并非单纯由原始淋巴细胞残留引起，而是可能源于多谱系造血细胞的受累，这种具有独特生物学特性的疾病亚型被定义为多谱系累及Ph^+^ ALL。目前，多谱系累及Ph^+^ ALL尚缺乏明确的诊断标准及预后评估体系，其临床治疗和患者管理面临诸多困难。因此，本文系统梳理了该亚型的分子病理特征、潜在预后标志物、动态演进规律及临床转归特点，旨在为制定更为精准的诊疗策略提供思路。

## Abstract (en)

The evolving stratified treatment approach based on molecular genetic alterations and minimal residual disease（MRD）monitoring has established a strong foundation for clinically managing Philadelphia chromosome-positive acute lymphoblastic leukemia（Ph^+^ ALL）. However, with the growing use of immune-targeted therapies and the increased sensitivity of detection technologies, discrepancies in MRD assessment have emerged in some patients with Ph^+^ ALL, particularly where BCR::ABL1-based MRD levels remain consistently elevated compared to those detected by alternative methods. Research suggests that this persistent BCR::ABL1 positivity may not solely reflect residual lymphoblasts but may also indicate the involvement of multilineage hematopoietic cells. This distinct biological feature has been termed Ph^+^ ALL with multilineage involvement. Currently, the absence of standardized diagnostic criteria and prognostic frameworks for this subtype poses significant challenges in clinical decision-making. Therefore, this article offers a comprehensive review of its molecular and pathological characteristics, potential prognostic biomarkers, patterns of disease evolution, and clinical implications, with the goal of informing more accurate diagnostic and therapeutic strategies.

Ph染色体阳性急性淋巴细胞白血病（Ph^+^ ALL）作为成人ALL的重要亚型，其发病率随年龄增长而递增。在儿童ALL患者中，Ph^+^ ALL的占比为2％～5％；在青少年及年轻成人ALL中约占6％；而在成人患者中，这一比例超过了25％[1]–[2]。传统观点认为，与慢性髓性白血病（CML）这类起源于多能造血干细胞且具有多谱系分化潜能的血液系统疾病不同，Ph^+^ ALL是局限于淋系定向细胞的克隆性疾病[3]–[6]。然而，随着分子检测技术的进步，Ph^+^ ALL的生物学异质性逐渐被揭示和报道。通过荧光原位杂交（FISH）、PCR及多参数流式细胞术（MFC）等技术的联合应用，多项研究发现，27％～43％的Ph^+^ ALL患者存在BCR::ABL1融合基因跨谱系扩散现象，表现为髓系等非淋巴系细胞的受累[7]–[10]。尽管此类患者常缺乏典型CML的临床特征（如高白细胞血症、BCR::ABL1阳性非肿瘤细胞高占比等），但是其BCR::ABL1阳性克隆性造血模式与CML高度相似。基于此，多谱系累及Ph^+^ ALL亚型被证实与确立[11]。这种生物学异质性在一定程度上增加了微小残留病（MRD）监测的难度，进而影响患者治疗方案的选择。为应对这一挑战，国内外诸多研究者对多谱系累及Ph^+^ ALL开展了广泛的基础及临床探索。本文将对其研究进展进行系统性梳理总结，旨在为多谱系累及Ph^+^ ALL临床诊疗策略的优化提供参考。

一、多谱系累及Ph^+^ ALL的特点及诊断标准

多谱系累及Ph^+^ ALL的发现源于部分Ph^+^ ALL患者在使用不同MRD检测方法时，出现了结果不一致的现象。这一差异引发了学术界对多谱系累及Ph^+^ ALL的广泛研究，但至今尚未就其明确的诊断标准达成国际统一共识。

早在2009年，Zaliova等[12]开展了一项针对17例接受BFM治疗方案的Ph^+^ ALL儿童患者的研究，其通过纵向实时定量PCR（RQ-PCR）检测发现，20％的样本免疫球蛋白/T细胞受体（IG/TR）检测呈阴性而BCR::ABL1检测呈阳性。随着酪氨酸激酶抑制剂（TKI）靶向治疗的应用，这一差异更加明显。伊马替尼联合化疗的研究显示，使用PCR技术分别检测BCR::ABL1和IG/TR重排结果的一致性仅为69％，BCR::ABL1重排的阳性率显著更高[13]。值得注意的是，这两项研究中IG/TR检测受V（D）J重组复杂性影响，其灵敏度阈值为10^−4^；而BCR::ABL1检测可实现更高的灵敏度（10^−5^），灵敏度差异可能在一定程度上解释了MRD检测结果的不一致性。

然而，竺晓凡团队在CCCG-ALL-2015研究[9]中揭示了更深层的机制。研究发现，即使在方法学灵敏度匹配的条件下，仍有27.1％的患者存在1个以上MRD差异>1log的样本，且均为PCR检测BCR::ABL1重排的定量值高于MFC检测结果。这一发现亦被其他研究所证实，在一项针对使用Hyper-CVAD联合TKI治疗的成人Ph^+^ ALL患者的研究中，虽经二代测序（NGS）检测IG/TR重排的灵敏度高达10^−6^，但仍有15％～30％的NGS阴性样本出现BCR::ABL1阳性[14]。这些研究证据表明，MRD检测结果的差异并非检测技术灵敏度所致。进一步的分子机制研究证实，在此类BCR::ABL1阳性率显著高于其他MRD检测方法的患者中，BCR::ABL1融合基因不仅存在于ALL肿瘤细胞，同时也出现在非ALL淋巴细胞、红细胞和髓系细胞中，这种多谱系累及现象可能是造成不同MRD检测方法结果差异的关键因素[9],[11],[15]–[16]。

针对BCR::ABL1多谱系累及的现象，美国国立综合癌症网络（NCCN）建议使用FISH方法检测粒细胞中的BCR::ABL1重排，以便区分两个亚型[17]。然而，在临床诊疗中，由于受到FISH无法精准区分细胞类型等诸多限制，其在实际应用中存在一定困难。鉴于这种情况，有研究者提出替代性诊断策略，即当不同方法检测MRD的结果不平行且出现于2个及以上的时间点时，可提示多谱系累及[11],[16]。如MFC-MRD阴性但PCR检测BCR::ABL1融合基因阳性（高于0.1％），或者MFC与PCR检测BCR::ABL1融合基因虽均为阳性，但PCR定量值显著高于MFC且差异大于1log。不过，上述策略仍存在多重局限性。首先，检验误差的干扰是一个不可忽视的问题，RT-qPCR存在±0.5log的固有变异系数，需参照EuroMRD联盟（European Research Initiative on MRD Standardization）等规范进一步形成标准化检测体系；其次，患者的异质性可能导致全部BCR::ABL1融合基因阳性细胞均被快速清除的患者出现诊断遗漏。因此，后续还需要进一步研究以实现BCR::ABL1累及细胞群的早期、准确、简便的区分，以指导临床诊断与治疗决策。

二、多谱系累及Ph^+^ ALL的临床和生物学特征

在诊断标准亟待优化的背景下，深入解析多谱系累及Ph^+^ ALL的生物学异质性成为突破现有困境的关键路径。

一项针对59例儿童Ph^+^ ALL的研究显示，多谱系累及亚型在性别、年龄、WBC、中枢神经系统（CNS）状态等基线特征上与仅淋系累及亚型之间的差异无统计学意义[9]。但基于成人患者的研究结论略有不同。Kim等[7]发现多谱系累及患者外周血中性粒细胞计数显著升高，且p210转录本比例更高。Kamoda等[18]进一步证实，多谱系累及组WBC显著高于仅淋系累及组［182.1（22.5～496.4）×10^9^/L对15.0（1.8～350.4）×10^9^/L，*P*＝0.006］，且常伴随髓系抗原（CD13/CD33）表达。上述差异提示，多谱系累及亚型可能起源于更早期细胞。

围绕以上发现，Kim等[19]通过多组学分析对BCR::ABL1阳性ALL的发育轨迹进行了研究，并鉴定出三种转录组亚型（早期祖B细胞、中间祖B细胞、晚期祖B细胞），其中多谱系累及患者主要分布于发育阶段更早的早期祖B细胞亚群。在此基础上，Bastian等[20]通过跨队列整合分析（327例BCR::ABL1阳性ALL患者）进一步取得突破。具体而言，他们创新性地整合了4个独立队列，采用了转录组测序、全基因组测序、流式细胞术分选FISH验证等多维度组学技术，发现Ph^+^ ALL存在两个具有显著生物学差异的转录组主簇（C1/C2）。C1簇（多谱系累及型）经FISH验证显示均存在髓系细胞BCR::ABL1阳性，其基因组特征表现为HBS1L基因座结构性缺失及7号染色体单体的高频发生（发生率分别为43.8％与35.4％），转录特征与祖B细胞（pro-B）高度相似，且伴随CD13/CD33等髓系抗原的高表达；而C2簇（仅淋系累及型）基因组层面以IKZF1双等位缺失及PAX5/CDKN2A共缺失为特征，高频率表达淋系抗原CD20及CD22，更接近pre-B I细胞。值得注意的是，HBS1L基因座缺失被证实为多谱系累及Ph^+^ ALL的核心协同事件，其诱导的HBS1Lalt转录本（长读长测序验证）通过调控造血干/祖细胞分化潜能及肿瘤细胞的转化阶段，可能成为多谱系累及亚型的分子鉴别标志，为临床精准分型提供了关键生物学依据。但是，由于缺乏国际共识性诊断标准且未能有效排除CML急淋变的干扰，当前分子分型体系仍需通过多中心大样本研究进一步完善。

三、多谱系累及Ph^+^ ALL的治疗及预后

TKI联合化疗/免疫治疗显著改善了Ph^+^ ALL的预后[21]–[26]，然而，多谱系累及亚型的独特生物学异质性对现行的Ph^+^ ALL临床管理策略提出了许多新的挑战，如何对多谱系累及的患者进行临床精细化管理并改善其远期预后仍是许多研究的重点。

首要的难点在于MRD结果的准确解读。在既往Ph^+^ ALL的临床治疗中，MRD结果被认为是风险评估和治疗决策调整的重要参考，MRD残留是预示患者预后不佳的重要因素，并提示可能需要进行异基因造血干细胞移植（allo-HSCT）等治疗策略的转变[27]–[30]。但是有研究指出，多谱系累及Ph^+^ ALL和仅淋系累及Ph^+^ ALL两种亚型的预后生物学标志物有所不同。在多谱系累及患者中，传统的基于BCR::ABL1的MRD检测模式可能无法准确评估分子残留风险，需通过多维生物标志物联合动态分析，如纳入流式细胞术、NGS等监测其他遗传学标志物来提升风险分层和预后评估的精准性[9],[14],[16]。

关于allo-HSCT适应证评估，学界对多谱系累及Ph^+^ ALL是否可从移植中获益仍存在争议。竺晓凡团队[9]在多谱系累及Ph^+^ ALL和仅淋系累及Ph^+^ ALL患者中，分别于诱导治疗的第19天和第46天、巩固治疗结束、再诱导治疗前、完成5个周期的持续治疗及治疗完成共六个时间点进行MRD评估，发现两组患者MFC-MRD阳性率在所有时间点差异均无统计学意义（*P*>0.05），且5年累计复发率（48.9％对38.0％，*P*＝0.31）及无事件生存率（49.2％对55.7％，*P*＝0.50）差异亦无统计学意义。这提示多谱系累及的儿童患者接受传统ALL方案治疗后可能无需allo-HSCT即可获得长期生存。这一发现与Zuna等[16]在儿童队列及Short等[14]在成人队列中的研究结论相似，多谱系累及和仅淋系累及ALL患者5年总生存率及无复发生存率差异均无统计学意义。值得关注的是，尽管预后相似，但多谱系累及亚组患者接受造血干细胞移植的比例显著更高，这为结论带来了一定偏倚。此外，Kim等[19]研究发现，在多谱系累及亚组中主要分布的早期祖B细胞亚群，其携带的HBS1L缺失亦与极差预后显著相关。同时，既往认为在Ph^+^ ALL患者中存在诸如IKZF1^plus^基因型等特殊高危因素[31]–[35]，这些高危因素在多谱系累及患者中的预后权重也尚未明确。因此，多谱系累及Ph^+^ ALL患者是否需要进行造血干细胞移植仍需更多前瞻性研究验证。

尽管国际上对多谱系累及Ph^+^ ALL进行allo-HSCT的适应证尚未达成共识，目前的数据提示其治疗反应模式可能区别于MRD阳性仅淋系累及Ph^+^ ALL及CML急淋变患者[16],[36]–[38]。这一差异的生物学基础可追溯至三者克隆起源层级的差异及协同驱动突变的影响，即BCR::ABL1重排发生的细胞阶段，以及前文提到的协同驱动分子改变介导的B细胞阻滞阶段的不同。因此，未来研究方向需聚焦于以下关键问题：①通过单细胞测序技术解析三者的克隆演化模式，明确BCR::ABL1重排的发生时序及其与协同驱动分子事件的动态互作；②建立基于发育阶段的预后分层模型，为指导造血干细胞移植适应证的优化提供方向。

此外，由于两者在复发模式上存在差别，其临床管理的侧重点亦不相同。仅淋系累及Ph^+^ ALL复发多源于耐药克隆增殖。而Nagel等[39]对两例贝林妥欧单抗（Blinatumomab）治疗后CD19阴性复发的Ph^+^ ALL成人患者追踪研究发现，两例患者在初次诊断时均存在多谱系累及，且其复发时IG基因表现为胚系构型而非初次诊断时存在的克隆性重排。这提示患者的CD19阴性复发是由CD19阴性的Ph^+^阳性祖细胞演变而来。据此，Nagel等推荐对该类患者联合应用TKI以靶向CD19阴性而BCR::ABL1阳性的祖细胞。与之类似，Zuna等[16]于2022年发表的研究中提出，鉴于BCR::ABL1阳性非ALL细胞可能的复发风险，推荐对持续BCR::ABL1阳性的患者在整个治疗期间及治疗结束后定期监测MRD，并且长期维持TKI治疗。然而，在后续的研究中，Zuna等[40]报道了1例儿童Ph^+^ ALL患者，该患者在经历了为期1年的伊马替尼治疗后，序贯进行了15个月达沙替尼治疗并最终停药，停药后该患者IG/TR检测呈阴性但BCR::ABL1持续处于低水平阳性状态。进一步研究表明，该患者的BCR::ABL1负荷仅在治疗最初时出现轻度的下降，此后无论是进一步延长TKI治疗或停药均未显著改变BCR::ABL1水平。基于此发现并考虑到TKI治疗的不良反应，他们更新了关于在多谱系累及的患者中长期使用TKI的建议，提出对于该类患者仅需每3～6个月检测外周血的BCR::ABL1水平，只要其BCR::ABL1负荷维持在低水平且没有明显的增加便无需延长TKI治疗，仅在病情进展或血液学复发时再恢复TKI给药或进行造血干细胞移植。Short等[14]在成人研究中也观察到BCR::ABL1负荷对治疗无反应的现象，与Zuna等在儿童患者中观察到的现象相似，他们发现在MRD不一致的患者中，包括进一步延长TKI治疗在内的各种治疗方法均对治疗后BCR::ABL1的低水平阳性状态没有显著影响，但是患者经由高灵敏度NGS检测IG/TR结果呈阴性，并且达到了持久的缓解和较长的生存。基于上述研究，目前并不推荐在多谱系累及Ph^+^ ALL患者中延长TKI的使用，但需定期检测其MRD水平以对可能的复发做出迅速的反应。然而，关于BCR::ABL1阳性的非肿瘤细胞是否存在克隆演化潜能，目前国际血液学界对此尚未达成统一共识，亟待开展多中心前瞻性研究阐明其生物学本质与临床管理策略。

综上所述，多谱系累及Ph^+^ ALL与仅淋系累及Ph^+^ ALL在MRD动态水平、克隆演化模式及临床预后等方面存在诸多不同。尽管二者均以BCR::ABL1融合基因为核心驱动事件，但其克隆起源和协同驱动基因的深层次差异可能造成分化阶段与临床转归的不同，而深入解析两类亚型的克隆演进机制或可成为阐明其本质差异的关键突破口。此外，当前的诊断体系亟待更新，建立基于多维生物标志物的早期诊断标准可以更好地识别不同亚型，具有重要的临床意义。通过这项框架的创新，有望为多谱系累及Ph^+^ ALL患者提供更加精准的预后分层和个性化的治疗方案，为该类患者临床诊疗奠定更加坚实的基础。
